# The evolution of public health statistical modeling approaches and how to advance their incorporation into modern arboviral surveillance

**DOI:** 10.1093/jme/tjaf127

**Published:** 2025-10-15

**Authors:** Maggie McCarter, Stella C W Self, Alex Ewing, Mufaro Kanyangarara, Sarah M Gunter, Melissa S Nolan

**Affiliations:** Department of Epidemiology and Biostatistics, Arnold School of Public Health, University of South Carolina, Columbia, SC, USA; Department of Epidemiology and Biostatistics, Arnold School of Public Health, University of South Carolina, Columbia, SC, USA; Data Support Core, Prisma Health, Greenville, SC, USA; Department of Epidemiology and Biostatistics, Arnold School of Public Health, University of South Carolina, Columbia, SC, USA; Department of Pediatrics, Baylor College of Medicine, Houston, TX, USA; Department of Epidemiology and Biostatistics, Arnold School of Public Health, University of South Carolina, Columbia, SC, USA

**Keywords:** Arbovirus, forecast modeling, Markov Chain Monte Carlo method, Integrated Nested Laplace Approximation technique, Bayesian statistics

## Abstract

Statistical modeling of infectious disease transmission patterns has been in existence since the mid-1700s, evolving in their utility as the scientific and technological revolutions progressed. Despite the expansion of emerging mathematical and statistical methodologies over the past 250 yr, their usage has largely remained restricted to academic settings. This forum article will discuss the evolution of disease modeling techniques, the most common types of models in use today, and recommendations on how key archetypes can be incorporated into future public health practice. With the recent global impetus to predict and forecast novel pathogens, this article raises the question: *Why are endemic arboviruses not included in public health modeling efforts, and how can medical entomologists promote their inclusion?*

## Graphical abstract

**Graphical Abstract:**
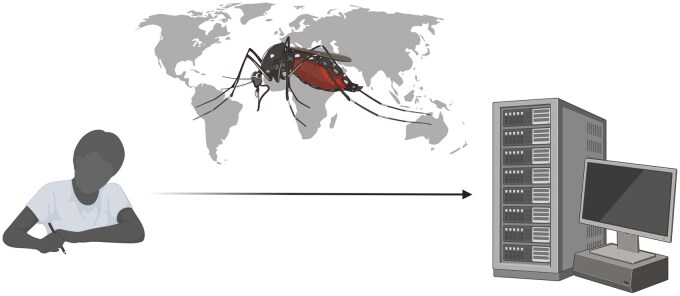

The SARS-CoV-2 pandemic highlighted the importance of mathematical models for public health professionals, as many sought to understand this novel pathogen and its likely trajectory. During the early and highly uncertain stages of the pandemic, governments and other stakeholders heavily relied on predictive modeling to identify at-risk areas and forecast infection rates to guide decision-making. While the pandemic resulted in abundant funding for SARS-CoV-2 modeling and led to the establishment of the US Centers for Disease Control and Prevention’s Center for Forecast and Outbreak Analytics, there remains little funding for disease forecasting of important arboviruses, such as West Nile virus (WNV), dengue virus (DENV), and La Crosse encephalitis virus (LACV) (Redfield 2020, Centers for Disease Control and Prevention Justification of Estimates for Appropriation Committees Fiscal Year 2025, Centers for Disease Control and Prevention Justification of Appropriation Estimates for Appropriations Committees Fiscal Year 2026). These diseases face chronic under-attention and recurrent cyclical outbreaks. The recent mathematical modeling renaissance begs the question: *Why is such an essential public health tool underutilized and underfunded for endemic diseases, such as WNV, DENV, and LACV?* Further, what steps can be taken to increase the integration of these evidence-based prediction tools into routine public health planning practice? This forum article’s objective is to highlight the inequities around vector-borne disease modeling and incite meaningful conversation to create policy-level changes for integration of forecast models into public health practice for proactive, evidence-based resource allocation. This 3-part article will discuss (i) the origin and evolution of infectious disease modeling, (ii) the fundamentals of mathematical and statistical models and their specific applicability, and (iii) recommendations to promote the acceleration of modeling techniques for vector-borne diseases and adoption of arboviral predictive models among public health professionals.

## History and Evolution of Infectious Disease Modeling

### Infectious Disease Mathematical Modeling in the Pre-Computer Era

Although mathematics as a discipline originated in ancient times, the first paper which applied mathematical modeling to infectious disease was not published until 1766 (Bernoulli 1766). The recognition of pathogens and the molecular basis of infection were emerging concepts during the transition between the Renaissance and the Scientific Revolution, affording Bernoulli the unique opportunity to combine an established discipline (mathematics) with an emerging one (infectious disease) (Adams 2021). Smallpox ravaged Europe and North America during the 18th century wars, and efforts were being made to control the disease. Swiss mathematician Daniel Bernoulli introduced a model to assess the effect of smallpox vaccination on smallpox mortality using a formula derived from children’s life expectancy at birth, lifetime infection risk, and smallpox virulence (Bernoulli 1766, Dietz and Heesterbeek 2002, Siettos and Russo 2013). He found that immunization against the disease increased the average life expectancy of children by 3 yr. His research, along with Edward Jenner’s research on vaccinations, led to the widespread acceptance of the smallpox vaccine (Jenner 1801). However, even though Bernoulli’s introduction to infectious disease mathematical modeling proved extremely useful, these methods did not become popular until the early 1900s, when mathematical epidemiology was first officially introduced by Ronald Ross and was utilized in a study with scientist George Macdonald (R. Ross 1905, S. R. Ross 1911).

The “Ross-Macdonald” transmission dynamics mathematical model was the first recorded arbovirus transmission model (Smith et al. 2012). This model defined a reproduction number, R_0_, for malaria as a function of susceptible versus infected individuals and interactions between individuals and mosquitos. The transmissibility of a pathogen, represented by R_0_, originally designated Z_0_, still plays an important role in modern prediction modeling. An R_0_ >1 indicates increasing malaria transmission, whereas an R_0_ ≤1 indicates stable or declining malaria transmission (Baum et al. 2020, Jin et al. 2020). Pyotr Enko later built models involving dynamics between infectious and susceptible individuals; Reed and Frost further described such models. These researchers first introduced the utilization of Markov chain methods for infectious disease (EN’KO 1989, Siettos and Russo 2013). The resulting surge of mathematics’ role in infectious disease was further catalyzed by the introduction of computers in the mid-1900s (EN’KO 1989, Siettos and Russo 2013).

### The Technological Revolution of Infectious Disease Modeling

The 1960s marked the proliferation of computer use in multiple disciplines. Historically, mathematical models, though advanced for their time, were limited in their scope. The widespread adoption of computer technology led to pronounced growth in infectious disease modeling, as computers allowed scientists and mathematicians to use advanced models otherwise nearly impossible to implement manually. This revolution led to the introduction of one of the more common means of fitting infectious disease models: the Markov chain Monte Carlo (MCMC) method. Although the Monte Carlo probabilistic sampling technique was first introduced during World War II, the MCMC algorithm advanced this technique in 1952 but was not widely used by statisticians until 1990 (Gelfand et al. 1990, Robert and Casella 2011). Unlike the standard Monte Carlo algorithm, which randomly samples directly from the probability distribution in question, MCMC sampling is an iterative process, whereby samples are drawn from a probability distribution, which depends on the previous set of sampled values, creating a “chain” of sampled values. Under appropriate conditions, the values in this chain converge to a sample from the target probability distribution. This method is used to predict outcomes based on existing data, which proved extremely useful in the modeling of diseases, such as HIV infections ­(Peterson et al. 1990, Lange et al. 1992, Wild et al. 1993). MCMC methods were increasingly used in infectious disease epidemiology to fit Bayesian regression models, as a supplement to traditional surveillance techniques (O’Neill et al. 2000).

### Modern Accessibility of Infectious Disease Modeling Computational Techniques

The late 1990s and early 2000s marked the beginnings of applied practitioners’ awareness of accessible infectious disease modeling. More interpretable autoregressive integrated moving average (ARIMA) models gained popularity in infectious disease prediction among epidemiologists. In 2009, researchers proposed the integrated nested Laplace approximation (INLA) technique to further reduce computational expense in modeling (Rue et al. 2009). The INLA technique is a streamlined approach to approximating Bayesian models and can often be used instead of the standard MCMC approach. The INLA technique assumes the latent structure of a model follows a Gaussian Markov random field and assumes that observations are independent given latent effects. Rather than sampling from the joint posterior distribution of a model, as MCMC does, the INLA technique approximates the marginal posterior distributions of the model parameters, greatly reducing computational expense (Lindgren and Rue 2015).

Although models like ARIMA and fitting techniques such as INLA were being increasingly utilized in infectious disease modeling, they were primarily carried out and interpreted by highly trained statisticians. Because of this, many predictive models were often seen as theoretical rather than practical public health tools. The emergence of the SARS-CoV-2 pandemic brought a renewed light to the importance of infectious disease modeling in public health practice. Government and private institutions had newfound dependence on these models to forecast the spread of the virus (Ray et al. 2020, ­Nikolopoulos et al. 2021, Nixon et al. 2022). However, as implementing and interpreting such models historically required training in advanced mathematics or statistics, those in public health without such backgrounds were often poorly equipped to make use of these models. Subsequently, advanced mathematicians/statisticians who previously focused primarily on the theoretical development of these models sought out contextual knowledge of virus spread and population interactions to make these predictive models useful for interpretation and application to public health practice (Ding et al. 2021). There is, therefore, a need for the merging of specialties in the broader health arena; public health scientists would benefit from a better understanding of the process and interpretations of infectious disease modeling, whereas mathematicians/­statisticians would benefit from a deeper knowledge of the ecology and transmission of pathogens.

## Modeling Fundamentals and Their Specific Disease System Applicabilities

To more broadly utilize infectious disease modeling in public health, it is imperative that public health professionals understand forecasting model basics, how they are applied, and how to interpret them (Lutz et al. 2019). Fundamental to this understanding is a recognition of the differences between the types of models themselves and the different model fitting procedures.

### Model Types

There are several families or classes of statistical models, which may fall into either the frequentist or the Bayesian paradigm. Many infectious disease forecasting models are regression models with distributional assumptions about the observed data. Models forecasting disease counts across time often use a Poisson or negative binomial distribution (Haight 1967). Conversely, models forecasting continuous variables such as mosquito population density frequently use linear regression or quantile regression procedures. Regression forecasting models are useful in assessing changes in model parameters and how such changes might affect the future. Such models can have a level of uncertainty in the estimates of the model parameters, in which appropriate measures of error should be accounted for (Davydenko and Fildes 2013). As spatial autocorrelation proves problematic in disease forecasting, especially in diseases such as arboviruses, spatio-temporal regression models have been developed to address such spatial autocorrelation (Legendre 1993). These models incorporate both temporal and spatial dependence in the model, allowing for area-specific disease forecasting. Multiple methods of estimating regression forecasting parameters exist; maximum likelihood estimation is the most common for frequentist models and MCMC, INLA, and maximum a posteriori estimations are popular for Bayesian models.

#### Spatiotemporal Models

Within the context of arboviruses, spatio-temporal regression models are heavily utilized, as researchers often seek to not only forecast these vector-borne diseases but to understand the spatial, environmental, and population features of increased disease rates in both humans and vectors. WNV and DENV are both modeled heavily by scientists exploring the relationships between disease incidence and various population, climatic, and environmental factors (Chancey et al. 2015, Marini et al. 2016, Butterworth et al. 2017, Murdock et al. 2017, Ryan et al. 2019, Maljkovic Berry et al. 2020, Keyel et al. 2021, Marinho et al. 2022, Holcomb et al. 2023, Harish et al. 2024). These relationships are not necessarily causal; nonetheless, quantifying how changes in the socio-demographic and environmental context of a disease correlate with changes in disease incidence can aid in prediction. Researchers have used Bayesian Poisson regression models, often estimated by the popular MCMC approach, to assess factors associated with disease incidence in multiple countries and found that factors, such as rainfall, temperature, and sociodemographic markers, all predicted dengue incidence (Phanitchat et al. 2019, Akter et al. 2021, Solís-Navarro et al. 2022). Other studies also utilized newer model fitting techniques such as INLA to assess factors associated with WNV incidence, with similar findings as the studies using MCMC fitting methods (Myer et al. 2017, Myer and Johnston 2019). Bayesian logistic regression models are frequently used to assess DENV vector presence/absence, as well as WNV vectors in certain areas (Hettiarachchige et al. 2018, Temple et al. 2022). Regression forecasting models are not currently used to predict LACV incidence, though recent attention given to infectious disease forecasting could instigate such research in the future (McCarter et al. 2025).

#### Compartmental Models

As opposed to regression models that assess model parameters and their influence on disease forecasts with distributional assumptions, compartmental models assess interactions between people, vectors, and disease dynamics, with no underlying distributional assumptions. These models are often used to predict disease spread, the number of infected individuals, and how long an epidemic is forecasted to last (Hethcote 1989). In compartmental models, individuals in a population are assigned to various “compartments”; the most basic 3 are “susceptible,” “infectious,” and “recovered.” This model, coined the SIR model, is the simplest and foundational compartmental model (Harko et al. 2014, Kröger and Schlickeiser 2020). However, as many diseases are unique in population and disease dynamics, other compartments have been added to account for these dynamics. Models such as the Susceptible Exposed Infectious Recovered (SEIR) model account for latency periods where an individual is infected but not yet infectious. Compartmental models can be used to assess the impact of hypothetical changes in transmission dynamics, such as reducing the number of susceptible individuals through vaccination or reducing the amount of contact between infectious and susceptible individuals.

Compartmental models can be paired with distributional assumptions to create either a Bayesian or frequentist statistical model or can be strictly mathematical with no uncertainty quantification. These models quantify the interactions and dynamics of each of these compartments using a system of differential (or difference) equations, which is solved to forecast disease incidence and spread. These dynamics are generally assumed to be causal; for example, an increase in the number of infected individuals will cause a subsequent increase in the number of recovered individuals. Compartmental models are heavily utilized in vector-borne disease forecasting. SIR and SEIR models have historically proven useful in predicting DENV infection rates (Newton and Reiter 1992, Esteva and Vargas 1998). Mathematical epidemiologists developed multiple models to assess WNV transmission between avian hosts, mosquito vectors, and humans (DeFelice et al. 2017, Angelou et al. 2021). A compartmental model predicting LACV $R_{0}$ in the Appalachia region of the United States assessed the dynamics in systems with *A. triseriatus* and *A. albopictus* and found that LACV transmission occurred in most models with tree-hole mosquitos as the primary vector (Bewick et al. 2016).

#### Agent-Based Models

Agent-based models are similar to compartmental models, but rather than dividing the population into compartments, they model each individual in the population separately (Technical Explainer: Infectious Disease Transmission Models 2025). These individuals (referred to as “agents”) are assigned a disease state (eg susceptible, infectious, or recovered) and other characteristics that may be relevant for transmission dynamics (eg age, occupation, etc.). The agents interact according to a set of prespecified rules, which govern the degree of interaction between individuals with different characteristics, the chances of infection spreading during interactions, and the duration of infection. When modeling arboviral diseases, the agents are generally a mixture of vectors, reservoir host animals and humans. Agent-based models tend to be computationally expensive and require a lot of data about the population of interest. Nevertheless, agent-based models have been successfully used to model DENV and, to a lesser extent, WNV (Jacintho et al. 2010, Nasrinpour et al. 2019, Perkins et al. 2019).

#### Machine-Learning Models

Machine-learning models are increasingly being utilized as high-performance computers are developed, and as surveillance datasets increase in size. Machine-learning models are a very broad class of statistical archetypes, which specify parameters for models to train based on past data and potentially improve these estimates when they encounter new future data (Mitchell and Mitchell 1997). While other models, such as regression models, make inferences on relationships between disease incidence and other cofactors, machine-learning techniques typically focus solely on the most accurate predictions possible with less emphasis on model interpretability or explainability. Like regression models, machine-learning models do not require causal relationships between the covariates and the outcome (disease incidence) but exploit the phenomenological relationships between these factors to predict disease incidence. These predictions are made by using a subset of data to “train” the model and then using the remaining data to evaluate the performance of the trained model. Many researchers recently utilized machine learning to accurately predict WNV and DENV outbreaks across the globe (Farooq et al. 2022, Nguyen et al. 2022, Roster et al. 2022, Tonks et al. 2022). Such models can be factor-based, often including environmental and climatic factors in their forecasting. However, the relationship between these factors and the disease in question is often not elucidated by a machine-learning model, and it may be difficult to ascertain how changes in these factors will influence disease incidence. Additionally, these methods are often extremely computationally expensive and require large datasets to be accurate; therefore, they are often reserved for contexts when access to high-performing computers is feasible.

#### Frequentist Versus Bayesian Modeling

Most infectious disease models can be categorized as either mathematical models or statistical models, with statistical models providing both estimates and uncertainty quantification (eg confidence intervals) and the mathematical models providing estimates only. Statistical models may be further classified as either frequentist or Bayesian. Frequentist statistics consider model parameters as fixed but unknown constants which are estimated from observed data. Uncertainty is typically quantified via confidence or prediction intervals derived from the sampling distribution of the model parameters; upon repeated sampling, the estimated interval captures the parameter of interest a certain pre-specified percentage of the time (often 95% is used). Within the frequentist analysis framework, researchers can perform hypothesis tests using *P*-values to quantify the probability of the observed event, or a “more extreme” event, occurring if the null hypothesis were true (Cox 2006). The frequentist framework is more familiar and widely used in various disciplines, as it requires fewer assumptions regarding the model parameters than its Bayesian counterpart.

Bayesian statistics derives its name from Bayes’ theorem, in which the probability of an event is directly related to previous knowledge of the parameters related to that event (Joyce 2003). For example, in forecasting WNV in the United States, models using Bayesian forecasting rely on prior established WNV knowledge such as previous infection rates to predict rates for the future (Myer and Johnston 2019). Thus, Bayesian statistics can be more useful for predicting or modeling endemic diseases versus novel or invasive vectors and their associated diseases. In the Bayesian framework, model parameters are random quantities with associated distributions; the prior distribution of the parameters reflects what is known about the parameters prior to observing any data. After observing data, Bayes theorem is used to update the prior distribution to obtain the posterior distribution. Rather than the confidence intervals produced from frequentist analyses, Bayesian inference often relies on credible intervals for model parameters. The typical interpretation of a Bayesian 95% credible interval is that given the observed data, there is a 95% probability that the parameter falls into the interval (Stone 2013, van de Schoot et al. 2021). Bayesian statistics is becoming more common in research, as it offers multiple advantages over frequentist frameworks. Bayesian analysis allows researchers to assess ranges of certainty of results within credible intervals rather than simply point estimates, as well as calculate probability distributions of said results (van de Schoot et al. 2021). Additionally, performing inference on complex models is often computationally easier and more reliable in the Bayesian paradigm.

## Expanding Arboviral Forecasting for Future Public Health Interventions

Currently, more than 500 arboviruses are recognized globally, 150 of which are known pathogens to humans, and others pose unknown human disease risks (Young 2018). Such arboviral diseases can cost governments and societies billions of dollars globally through decreased tourism, impaired workforce capacity, and aggregate resident healthcare and disability costs (Thompson et al. 2020). However, even with their high prevalence and clinical and economic impact, there are very few resources allocated to arboviral surveillance, forecasting, and prevention globally; existing initiatives receive little funding from governments and institutions. For example, since 2022, approximately 10% (range 6% to 11%) of the US Centers for Disease Control and Prevention’s Emerging and Zoonotic Infectious Diseases’ budget has been allocated for arboviral prevention, response, and research (Walensky 2022, 2024, Centers for Disease Control and Prevention Justification of Estimates for Appropriation Committees Fiscal Year 2025). For fiscal year 2026, the American President requested $870,486,000 for the US Centers for Disease Control and Prevention to combat emerging and zoonotic infectious diseases, of which $87,817,000 for vector-borne diseases (Centers for Disease Control and Prevention Justification of Appropriation Estimates for Appropriations Committees Fiscal Year 2026). The extent that this funding goes toward modeling is unknown; yet, the Vector-borne Disease Division does support the annual maintenance of ArboNet and TickNet, for local jurisdictions to report their human, veterinary, and vector cases of select pathogens creating a centralized database for potential modeling efforts (Centers for Disease Control and Prevention Justification of Appropriation Estimates for Appropriations Committees Fiscal Year 2026). Similarly, from January 2020 to July 2025, the National Institutes of Health’s eREPORTER database lists 102 grant funded records accumulating $50,328,405 in SARS-COV-2 related modeling research compared to 13 arboviral modeling associated grant funded records accumulating $9,911,490.

Another likely aspect contributing to underfunding of arboviral forecast modeling is their reliance on high-quality ecological studies and computational biology, which are often neglected in funding due to their niche focuses that misalign with federal funding agencies. For example, the US National Institutes of Health prioritizes medical research, the National Science Foundation prioritizes non-health-related scientific discovery, and the Centers for Disease Control and Prevention prioritizes public health programs. However, arboviral prediction modeling is not purely medical (it requires vector ecology inputs), not purely entomological (humans serve as hosts), and not directly applied public health work (theoretical advancements are critical in early model development), rendering this work misaligned to national funding agencies’ priorities, respectively. Promotion of this work could be facilitated by more funding for cross-agency infectious disease modeling specifically (eg the Ecology and Evolution of Infectious Diseases grant mechanism jointly sponsored by the US National Institutes of Health and National Science Foundation) or collaborative case competitions.

As research and public health funding wanes for arboviral prediction modeling, employment demands lag for a competent workforce, yielding an insidiously widening gap. The US Centers for Disease Control and Prevention recently initiated workforce development programs to mitigate medical entomology-related workforce shortages, including arboviral modelers; however, these programs are congressionally appropriated, leaving their funding subjective to current political sentiment. Historically, funding for these programs, such as the Centers of Excellence in Vector-borne Diseases, Regional Training and Evaluation Centers and Public Health Entomology for All, has had bipartisan support; however, shifting public health priorities often render the future of these workforce training programs uncertain (Senator King 2017, Senator Collins 2019, Dye-Braumuller et al. 2022).

The underfunding of such programs is also a narrative on health equity, as arboviruses disproportionately affect people living in poverty. Studies have shown populations with low socioeconomic status markers such as lower education, lower income, and poor housing are at increased arboviral infection risk (Power et al. 2022). Locations with rapidly increasing urbanization and human migration, typically associated with lower socioeconomic factors, are at the highest arboviral disease transmission risk (Tajudeen et al. 2021). These inequities in arbovirus infections highlight the need for better surveillance and forecasting methods, so stakeholders, governments, and institutions can recognize the need to allocate funds for at-risk, poverty-stricken areas. However, such forecasting methods are often expensive, requiring advanced computers and highly trained mathematicians or statisticians to run many models. Alternatives to such computationally expensive model approximations, such as the INLA method, implemented by the freely available, user-friendly R-INLA package, have great potential to make disease forecasting more accessible to areas where resources are limited (Lindgren and Rue 2015). However, the development of these methods alone cannot resolve the issue of under-surveillance and arbovirus forecasting in impoverished areas. Thus, there is a great need for better data availability in such areas to supplement these forecasting models to explore the relationships between arboviral infection and economic, population, and environmental factors. Increased knowledge of both the context of infectious disease and the application of mathematical and statistical models can bridge the gap between advanced statisticians and public health officials and thus build the foundation of resolving the problem of inequity in the exploration of arboviruses globally.

## References

[tjaf127-B1] Adams DP. 2021. Foundations of infectious disease: a public health perspective. Jones & Bartless Publishers.

[tjaf127-B2] Akter R , Hu W, Gatton M, et al 2021. Climate variability, socio-­ecological factors and dengue transmission in tropical Queensland, Australia: a Bayesian spatial analysis. Environ. Res. 195:110285. 10.1016/j.envres.2020.110285. 33027631

[tjaf127-B3] Angelou A , Kioutsioukis I, Stilianakis NI. 2021. A climate-dependent spatial epidemiological model for the transmission risk of West Nile virus at local scale. One Health 13:100330. 10.1016/j.onehlt.2021.100330. 34632040 PMC8493582

[tjaf127-B4] Baum J , Pasvol G, Carter R. 2020. The R0 journey: from 1950s malaria to COVID-19. Nature 582:488–488. 10.1038/d41586-020-01882-9. 32577000

[tjaf127-B5] Bernoulli D. 1766. Essai D’une nouvelle analyse de la mortalite cause par la petite verole et des a vantages de Finoculation pour la prevenir. Mem Math. & Phys. De I Acad. Roy. Sci. Hist. de I Acad. Paris 1.

[tjaf127-B6] Bewick S , Agusto F, Calabrese JM, et al 2016. Epidemiology of La Crosse Virus Emergence, Appalachia Region, United States. Emerg. Infect. Dis. 22:1921–1929. 10.3201/eid2211.160308. 27767009 PMC5088026

[tjaf127-B7] Butterworth MK , Morin CW, Comrie AC. 2017. An analysis of the potential impact of climate change on dengue transmission in the Southeastern United States. Environ. Health Perspect. 125:579–585. 10.1289/ehp218. 27713106 PMC5381975

[tjaf127-B8] Centers for Disease Control and Prevention Justification of Appropriation Estimates for Appropriations Committees Fiscal Year 2026. Atlanta, GA: Centers for Disease, Control and Prevention.

[tjaf127-B9] Centers for Disease Control and Prevention Justification of Estimates for Appropriation Committees Fiscal Year 2025.

[tjaf127-B10] Chancey C , Grinev A, Volkova E, et al 2015. The global ecology and epidemiology of West Nile virus. Biomed Res. Int. 2015:376230. 10.1155/2015/376230. 25866777 PMC4383390

[tjaf127-B11] Cox DR. 2006. Principles of statistical inference. Cambridge University Press.

[tjaf127-B12] Davydenko A , Fildes R. 2013. Measuring forecasting accuracy: the case of judgmental adjustments to SKU-level demand forecasts. Int. J. Forecast. 29:510–522. 10.1016/j.ijforecast.2012.09.002.

[tjaf127-B13] DeFelice NB , Little E, Campbell SR, et al 2017. Ensemble forecast of human West Nile virus cases and mosquito infection rates. Nat. Commun. 8:14592. 10.1038/ncomms14592. 28233783 PMC5333106

[tjaf127-B14] Dietz K , Heesterbeek JAP. 2002. Daniel Bernoulli’s epidemiological model revisited. Math. Biosci. 180:1–21. 10.1016/S0025-5564(02)00122-0. 12387913

[tjaf127-B15] Ding C , Liu X, Yang S. 2021. The value of infectious disease modeling and trend assessment: a public health perspective. Expert Rev. Anti. Infect. Ther. 19:1135–1145. 10.1080/14787210.2021.1882850. 33522327

[tjaf127-B16] Dye-Braumuller KC , Gordon JR, McCoy K, et al 2022. Riding the Wave: reactive vector-borne disease policy renders the United States vulnerable to outbreaks and insecticide resistance. J. Med. Entomol. 59:401–411. 10.1093/jme/tjab219. 35064260 PMC8924968

[tjaf127-B17] EN’KO PD. 1989. On the course of epidemics of some infectious diseases. Int. J. Epidemiol. 18:749–755. 10.1093/ije/18.4.749. 2695472

[tjaf127-B18] Esteva L , Vargas C. 1998. Analysis of a dengue disease transmission model. Math. Biosci. 150:131–151. 10.1016/s0025-5564(98)10003-2. 9656647

[tjaf127-B19] Farooq Z , Rocklöv J, Wallin J, et al 2022. Artificial intelligence to predict West Nile virus outbreaks with eco-climatic drivers. Lancet Reg. Health. Eur. 17:100370. 10.1016/j.lanepe.2022.100370. 35373173 PMC8971633

[tjaf127-B20] FY 2023 | Congressional Justifications | Budget | CDC. 2022, March 28. https://www.cdc.gov/budget/fy2023/congressional-justification.html.

[tjaf127-B21] Gelfand AE , Hills SE, Racine-Poon A, et al 1990. Illustration of Bayesian inference in normal data models using Gibbs sampling. J. Am. Stat. Assoc. 85:972–985. 10.2307/2289594.

[tjaf127-B22] Haight FA. 1967. Handbook of the Poisson distribution. Wiley.

[tjaf127-B23] Harko T , Lobo FSN, Mak MK. 2014. Exact analytical solutions of the susceptible-infected-recovered (SIR) epidemic model and of the SIR model with equal death and birth rates. Appl. Math. Comput. 236:184–194. 10.1016/j.amc.2014.03.030.

[tjaf127-B24] Harish V , Colón-González FJ, Moreira FRR, et al 2024. Human movement and environmental barriers shape the emergence of dengue. Nat. Commun. 15:4205. 10.1038/s41467-024-48465-0. 38806460 PMC11133396

[tjaf127-B25] Hethcote HW. (1989). Three basic epidemiological models. In: SA Levin, TG Hallam, LJ Gross, editors. Applied mathematical ecology. Springer. p. 119–144. 10.1007/978-3-642-61317-3_5.

[tjaf127-B26] Hettiarachchige C , von Cavallar S, Lynar T, et al 2018. Risk prediction system for dengue transmission based on high resolution weather data. PLoS One 13:e0208203. 10.1371/journal.pone.0208203. 30521550 PMC6283552

[tjaf127-B27] Holcomb KM , Mathis S, Staples JE, et al 2023. Evaluation of an open forecasting challenge to assess skill of West Nile virus neuroinvasive disease prediction. Parasit. Vectors 16:11. 10.1186/s13071-022-05630-y. 36635782 PMC9834680

[tjaf127-B28] Jacintho LFO , Batista AFM, Ruas TL, et al 2010. An agent-based model for the spread of the dengue fever: a swarm platform simulation approach. Paper presented at: proceedings of the 2010 spring simulation multiconference. Society for Computer Simulation International; Orlando, Florida.

[tjaf127-B29] Jenner E. 1801. On the origin of the vaccine inoculation. Med. Phys. J. 5:505–508. PMC5598692 30489837

[tjaf127-B30] Jin X , Jin S, Gao D. 2020. Mathematical analysis of the Ross–Macdonald Model with quarantine. Bull. Math. Biol. 82:47. 10.1007/s11538-020-00723-0. 32242279 PMC7117789

[tjaf127-B31] Joyce J. 2003. *Bayes’ Theorem*. Accessed January 1, 2025. https://plato.stanford.edu/archives/spr2019/entries/bayes-theorem/.

[tjaf127-B32] Keyel AC , Gorris ME, Rochlin I, et al 2021. A proposed framework for the development and qualitative evaluation of West Nile virus models and their application to local public health decision-making. PLoS Negl. Trop. Dis. 15:e0009653. 10.1371/journal.pntd.0009653. 34499656 PMC8428767

[tjaf127-B33] Kröger M , Schlickeiser R. 2020. Analytical solution of the SIR-model for the temporal evolution of epidemics. Part A: time-independent reproduction factor. J. Phys. A: Math. Theor. 53:505601. 10.1088/1751-8121/abc65d.

[tjaf127-B35] Lange N , Carlin BP, Gelfand AE. 1992. Hierarchical Bayes models for the progression of HIV infection using longitudinal CD4 T-cell numbers. J. Am. Stat. Assoc. 87:615–626. 10.1080/01621459.1992.10475258.

[tjaf127-B36] Legendre P. 1993. Spatial autocorrelation: trouble or new paradigm? Ecology 74:1659–1673. 10.2307/1939924.

[tjaf127-B37] Lindgren F , Rue H. 2015. Bayesian spatial modelling with R-INLA. J. Stat. Soft. 63:1–25. 10.18637/jss.v063.i19.

[tjaf127-B38] Lutz CS , Huynh MP, Schroeder M, et al 2019. Applying infectious disease forecasting to public health: a path forward using influenza forecasting examples. BMC Public Health 19:1659. 10.1186/s12889-019-7966-8. 31823751 PMC6902553

[tjaf127-B39] Maljkovic Berry I , Rutvisuttinunt W, Sippy R, et al 2020. The origins of dengue and chikungunya viruses in Ecuador following increased migration from Venezuela and Colombia. BMC Evol. Biol. 20:31. 10.1186/s12862-020-1596-8. 32075576 PMC7031975

[tjaf127-B40] Marinho RdSS , Duro RLS, Mota MTdO, et al 2022. Environmental Changes and the Impact on the Human Infections by Dengue, Chikungunya and Zika Viruses in Northern Brazil, 2010–2019. IJERPH. 19: 12665. 10.3390/ijerph191912665. 36231964 PMC9566075

[tjaf127-B41] Marini G , Poletti P, Giacobini M, et al 2016. The role of climatic and density dependent factors in shaping mosquito population dynamics: the case of *Culex pipiens* in Northwestern Italy. PLoS One 11:e0154018. 10.1371/journal.pone.0154018. 27105065 PMC4841511

[tjaf127-B42] McCarter M , Self SCW, Li H, et al 2025. Validating a Bayesian spatio-temporal model to predict La Crosse virus human incidence in the Appalachian Mountain Region, USA. Microorganisms 13:812. 10.3390/microorganisms13040812. PMC12029143 40284648

[tjaf127-B43] Mitchell TM , Mitchell TM. 1997. Machine learning (Vol. 1, Issue 9). McGraw-Hill New York.

[tjaf127-B44] Murdock CC , Evans MV, McClanahan TD, et al 2017. Fine-scale variation in microclimate across an urban landscape shapes variation in mosquito population dynamics and the potential of Aedes albopictus to transmit arboviral disease. PLoS Negl. Trop. Dis. 11:e0005640. 10.1371/journal.pntd.0005640. 28558030 PMC5466343

[tjaf127-B45] Myer MH , Campbell SR, Johnston JM. 2017. Spatiotemporal modeling of ecological and sociological predictors of West Nile virus in Suffolk County, NY, mosquitoes. Ecosphere (Washington, D.C) 8:e01854. 10.1002/ecs2.1854. 30147987 PMC6104833

[tjaf127-B46] Myer MH , Johnston JM. 2019. Spatiotemporal Bayesian modeling of West Nile virus: identifying risk of infection in mosquitoes with local-scale predictors. Sci. Total Environ. 650:2818–2829. 10.1016/j.scitotenv.2018.09.397. 30373059 PMC7676626

[tjaf127-B47] Nasrinpour HR , Friesen MR, McLeod RD. 2019. Agent based modelling and West Nile Virus: a survey. J. Med. Biol. Eng. 39:178–183. 10.1007/s40846-018-0396-8.

[tjaf127-B48] Newton EA , Reiter P. 1992. A model of the transmission of dengue fever with an evaluation of the impact of ultra-low volume (ULV) insecticide applications on dengue epidemics. Am. J. Trop. Med. Hyg. 47:709–720. 10.4269/ajtmh.1992.47.709. 1361721

[tjaf127-B49] Nguyen KH , Anneser E, Toppo A, et al 2022. Disparities in national and state estimates of COVID-19 vaccination receipt and intent to vaccinate by race/ethnicity, income, and age group among adults ≥ 18 years, United States. Vaccine 40:107–113. 10.1016/j.vaccine.2021.11.040. 34852946 PMC8598948

[tjaf127-B50] Nikolopoulos K , Punia S, Schäfers A, et al 2021. Forecasting and planning during a pandemic: COVID-19 growth rates, supply chain disruptions, and governmental decisions. Eur. J. Oper. Res. 290:99–115. 10.1016/j.ejor.2020.08.001. 32836717 PMC7413852

[tjaf127-B51] Nixon K , Jindal S, Parker F, et al 2022. Real-time COVID-19 forecasting: challenges and opportunities of model performance and translation. Lancet. Digit. Health 4:e699–e701. 10.1016/S2589-7500(22)00167-4. 36150779 PMC9499327

[tjaf127-B52] O’Neill PD , Balding DJ, Becker NG, et al 2000. Analyses of infectious disease data from household outbreaks by Markov chain Monte Carlo methods. J. R. Stat. Soc. Ser. C Appl. Stat. 49:517–542.

[tjaf127-B53] Perkins TA , Reiner Jr, Jr., España G, et al 2019. An agent-based model of dengue virus transmission shows how uncertainty about breakthrough infections influences vaccination impact projections. PLoS Comput. Biol. 15:e1006710. 10.1371/journal.pcbi.1006710. 30893294 PMC6443188

[tjaf127-B54] Peterson D , Willard K, Altmann M, et al 1990. Monte Carlo simulation of HIV infection in an intravenous drug user community. JAIDS 3:1086. 2213509

[tjaf127-B55] Phanitchat T , Zhao B, Haque U, et al 2019. Spatial and temporal patterns of dengue incidence in northeastern Thailand 2006–2016. BMC Infect. Dis. 19:743. 10.1186/s12879-019-4379-3. 31443630 PMC6708185

[tjaf127-B56] Power GM , Vaughan AM, Qiao L, et al 2022. Socioeconomic risk markers of arthropod-borne virus (arbovirus) infections: a systematic literature review and meta-analysis. BMJ Glob. Health 7:e007735. 10.1136/bmjgh-2021-007735. PMC9014035 35428678

[tjaf127-B57] Ray EL , Wattanachit N, Niemi J, et al 2020, Consortium, on behalf of the C.-19 F. H. *Ensemble Forecasts of Coronavirus Disease 2019 (COVID-19) in the U.S*. (p. 2020.08.19.20177493). medRxiv. 10.1101/2020.08.19.20177493.

[tjaf127-B58] Redfield RF. 2020. Centers for Disease Control and Prevention Justification of Appropriation Estimates for Appropriations Committees Fiscal Year 2021.

[tjaf127-B59] Robert C , Casella G. 2011. A short history of Markov Chain Monte Carlo: subjective recollections from incomplete data. Statist. Sci. 26:102–115. 10.1214/10-STS351.

[tjaf127-B60] Ross R. 1905. The logical basis of the sanitary policy of mosquito reduction. Science (New York, N.Y.) 22:689–699. 10.1126/science.22.570.689. 17729473

[tjaf127-B61] Ross SR. 1911. The prevention of malaria. John Murray.

[tjaf127-B62] Roster K , Connaughton C, Rodrigues FA. 2022. Machine-learning-based forecasting of dengue fever in Brazilian cities using epidemiologic and meteorological variables. Am. J. Epidemiol. 191:1803–1812. 10.1093/aje/kwac090. 35584963

[tjaf127-B05669827] Rue H , Martino S, Chopin N. 2009. Approximate Bayesian Inference for Latent Gaussian Models. The Norwegian University for Science and Technology.

[tjaf127-B63] Ryan SJ , Carlson CJ, Mordecai EA, et al 2019. Global expansion and redistribution of *Aedes*-borne virus transmission risk with climate change. PLoS Negl. Trop. Dis. 13:e0007213. 10.1371/journal.pntd.0007213. 30921321 PMC6438455

[tjaf127-B64] Senator Collins SM. 2019. ‘S.1657. US Congress.

[tjaf127-B65] Senator King AS. 2017. ‘S. 849. US Congress.

[tjaf127-B66] Siettos CI , Russo L. 2013. Mathematical modeling of infectious disease dynamics. Virulence 4:295–306. 10.4161/viru.24041. 23552814 PMC3710332

[tjaf127-B67] Smith DL , Battle KE, Hay SI, et al 2012. Ross, macdonald, and a theory for the dynamics and control of mosquito-transmitted pathogens. PLoS Pathog. 8:e1002588. 10.1371/journal.ppat.1002588. 22496640 PMC3320609

[tjaf127-B68] Solís-Navarro M , Vargas-De-León C, Gúzman-Martínez M, et al 2022. A Bayesian prediction spatial model for confirmed dengue cases in the state of Chiapas, Mexico. J. Trop. Med. 2022:1971786. 10.1155/2022/1971786. 35664923 PMC9159893

[tjaf127-B69] Stone J. 2013. *Bayes’ rule: a tutorial introduction to Bayesian analysis*. 10.13140/2.1.1371.6801.

[tjaf127-B70] Tajudeen YA , Oladunjoye IO, Mustapha MO, et al 2021. Tackling the global health threat of arboviruses: an appraisal of the three holistic approaches to health. Health Promot. Perspect. 11:371–381. 10.34172/hpp.2021.48. 35079581 PMC8767080

[tjaf127-B71] Technical Explainer: Infectious Disease Transmission Models. 2025. US Centers for Disease Control and Prevention.

[tjaf127-B72] Temple SD , Manore CA, Kaufeld KA. 2022. Bayesian time-varying occupancy model for West Nile virus in Ontario, Canada. Stoch. Environ. Res. Risk Assess. 36:2337–2352. 10.1007/s00477-022-02257-4.

[tjaf127-B73] Thompson R , Martin Del Campo J, Constenla D. 2020. A review of the economic evidence of *Aedes*-borne arboviruses and *Aedes*-borne arboviral disease prevention and control strategies. Expert Rev. Vaccines 19:143–162. 10.1080/14760584.2020.1733419. 32077343

[tjaf127-B74] Tonks A , Harris T, Li B, et al 2022. *Forecasting West Nile Virus with Graph Neural Networks: Harnessing Spatial Dependence in Irregularly Sampled Geospatial Data* (arXiv : 2212.11367). arXiv. 10.48550/arXiv.2212.11367, preprint: not peer reviewed. PMC11220409 38962698

[tjaf127-B75] van de Schoot R , Depaoli S, King R, et al 2021. Bayesian statistics and modelling. Nat. Rev. Methods Primers 1. 10.1038/s43586-020-00001-2.

[tjaf127-B77] Walensky RP. 2022. Centers for Disease Control and Prevention Justification of Appropriation Estimates for Appropriations Committees Fiscal Year 2022.

[tjaf127-B78] Walensky RP. 2024. Centers for Disease Control and Prevention Justification of Appropriation Estimates for Appropriations Committees Fiscal Year 2024.

[tjaf127-B79] Wild P , Commenges D, Etcheverry B. 1993. A hierarchical Bayesian approach to the back-calculation of numbers of HIV-infected subjects. J. R. Stat. Soc. Ser. D. (The Statistician ) 42:405–414. 10.2307/2348474.

[tjaf127-B80] Young PR. 2018. Arboviruses: a family on the move. Adv. Exp. Med. Biol. 1062:1–10. 10.1007/978-981-10-8727-1_1. 29845521

